# The Swedish Covid-19 strategy and voluntary compliance: Failed securitisation or constitutional security management?

**DOI:** 10.1017/eis.2021.26

**Published:** 2021-10-19

**Authors:** Oscar Leonard Larsson

**Affiliations:** Swedish Defence University, Stockholm, Sweden

**Keywords:** Covid-19, Pandemic, Securitisation, Logic-of-Appropriateness, Swedish Corona Strategy

## Abstract

The Covid-19 pandemic that emerged in the spring of 2020 caused severe political, social, and economic turmoil throughout the world. In spite of early warning signals from the World Health Organization, countries struggled to shape their policy responses and countermeasures for curtailing the spread of the virus while also minimising the damage that any restrictions would inflict on the health and well-being of society at large. While some countries have adopted strict regulations and extraordinary measures after declaring ‘states of exception’ and ‘national emergencies’, others have relied upon expert recommendations and individual responsibility. Sweden is viewed as having adopted one of the latter type of approaches in that it places the responsibility for social distancing upon the individual. Is this an instance of a failed ‘securitisation’ process, or rather a sensible constitutional and political response to a severe security event? This article presents an in-depth analysis of the Swedish strategy for coping with Covid-19, arguing that this case illustrates that security management in a democratic state should direct greater attention to rule following in accordance with a logic of appropriateness rather than the rule breaking envisaged by securitisation theory.

## Introduction

Security scholars have defined pandemics as a specific type of security threat that concerns human health on individual, national, and global levels.^1^ Such security threats do not always call for traditional and sovereign countermeasures, such as securing territory and borders, but may instead require control of public movement. This has clearly been visible during the Covid-19 pandemic.^2^ The ramifications of this pandemic illustrate how outbreaks of transmittable diseases give rise to substantial political, economic, and social challenges as societies do their utmost to stop, or at least hinder, the spread of the disease. The various countermeasures employed may also have a substantial negative effect upon public health in general in addition to negative gendered effects upon women in particular.^3^ Furthermore, the emergence of complex social and political concerns regarding control and regulation illustrates how individual freedom can be compromised when nations ‘securitise’ pandemics. This indirectly threatens both individual health and the health of the population as a whole.

Securitisation theory has comprised a common approach for analysing security issues. This theory maintains that the employment of extraordinary measures is the main indicator that a given security issue has been acknowledged as a serious threat.^4^ However, different states frequently have different constitutional and organisational structures for coping with crises and security issues, and neither the willingness, nor even the possibility to overstep established rules and procedures in order to counter threats towards lives and fundamental values, are self-evident. For example, democratic societies face profound problems in respect to security threats in that security management is expected to not violate individual rights and existing constitutional frameworks. Scholars have nonetheless argued that a form of securitisation and crisis communication that successfully conveys the message of urgency, risk to individual lives, and threat to both nation and society would render exceptional measures possible. This view has been used to explain why certain democratic countries have imposed stricter control of borders, populations, and individuals through the implementation of such policies as lockdowns and/or curfews during the Covid-19 pandemic.^5^

Andrew Neal, who holds a contrasting position, argues that viewing security issues as extra-political in character leads us into a ‘Hobbesian trap’, such that security always stands in opposition to a civil body politic, individual rights, and constitutional/democratic national rule.^6^ Moreover, the theoretical assumption that successful securitisation implies the use of exceptional measures would blind us to how security issues could be managed and resolved within existing political limits upon sovereign and executive powers. Stated otherwise, securitisation, which entails speech acts and subsequent crisis communication, does not necessarily render exceptional measures possible. Not only may security issues be normalised, democratic states may simply choose to not violate constitutional or procedural rules when they address security issues. Although securitisation theory in the strict sense would imply that not violating established rules and procedures in response to a threat constitutes an instance of ‘failed securitisation’, an alternative view of the same situation would be that governments and security experts do not wish to violate the accepted rules of the game since this would be illegitimate within a democratic context.

All countries have sought to develop and implement policies that would make it possible to control the outbreak of Covid-19 at the national level. They have nonetheless responded quite differently to the threat in spite of a relatively shared understanding of the dangers of the Covid-19 pandemic, including how the virus is transmitted. The Swedish Covid-19 strategy has been controversial. Some have criticised it for overlooking individual deaths and accepting high mortality rates among the population as a whole in order to protect the economy insofar as the country avoided a large-scale shutdown of society.^7^^,^^8^ Others have claimed that Sweden deliberately aimed at herd immunity.^9^ Still others have expressed the belief that such more tempered measures may be sustainable in the long run.^10^

The key issue I wish to analyse concerns the underpinnings of the Swedish Covid-19 strategy. This will serve to explain how and why individual responsibility and voluntary compliance with recommendations came to play such key roles in efforts to counter the pandemic. It will also facilitate explaining the Swedish ‘failure to securitise’ the Covid-19 pandemic. These issues taken together comprise the primary research question addressed in the present article. My principal argument is that the Swedish Covid-19 strategy has resulted from the fact that the Swedish government and responsible public authorities did not wish to break established rules or ignore accepted procedures, regardless of the serious security threat posed by Covid-19. It is this that constituted a case of supposed failed securitisation in terms of securitisation theory. Following existing laws, organisational rules, and constitutional rights, including the advancement of individual responsibility, thus led to what has been viewed as weak management, with mortality rates higher than in other Nordic countries, although they are not necessarily higher than in other European states. Furthermore, the present study also reveals that the underlying premise of securitisation theory may in fact lead us astray in that it overly emphasises rule breaking while underestimating *rule following* in efforts to explain responses to security threats in democratic states.

## Background and previous research on Covid-19 responses

The implications of Covid-19 for global health are staggering, with over 4.6 million confirmed deaths by mid-September 2021. The Covid-19 virus outbreak was first reported on 31 December 2019, when the Chinese authorities notified the World Health Organization (WHO) China Country Office of several cases of pneumonia of unknown etiology in Wuhan City, Hubei Province that had significant respiratory complications. The WHO published its first comprehensive package of guidance documents for how countries should manage this previously unknown disease on 10 January.^11^ After it became known that human-to-human transmission had occurred, the WHO issued a Public Health Emergency of International Concern (PHEIC) on 30 January, which marked the sixh time the WHO had done so since the International Health Regulations (IHR) came into force in 2005. A WHO situation report noted a total of 7,818 confirmed cases worldwide that same day. It took another two months for Covid-19 to be characterised as a global pandemic.^12^

Flattening the curve^13^ quickly became an overarching goal for most countries that involved a range of differing regulations and policies. Efforts to ensure social distancing assumed paramount importance – from those enforced through general states of exception, lockdowns, curfews, school closures, and restrictions placed upon specific businesses, such as restaurants and pubs, to those that relied upon recommendations coupled with an acceptance of individual responsibility.

Early studies of how countries sought to address the dangers presented by Covid-19 have highlighted the specific emergency and crisis responses of individual states. For example, authoritative governments with a high level of risk-aversion that enjoy strong executive power can impose restrictions upon individual mobility and freedom quite quickly when deemed necessary.^14^ China's lockdown in Wuhan bears witness to the potential effectiveness of authoritative governments in coping with security issues. Furthermore, a comparison of countries in respect to mortality rates per capita suggests that certain states in Asia that had recently experienced pandemics were also quicker to act on alerts from the WHO concerning Covid-19.^15^ This has been confirmed by a South Korea case study regarding a quick response to a given Covid-19 outbreak and the use of finely tuned policy instruments for tracing and isolation.^16^

The Norwegian government, by employing the slogan ‘working together’, was able to create a supportive and cohesive culture that served to increase its overall governance capacity. Creating consensus on what the crisis involved and what was necessary to effectively address it helps explains the measures they implemented to control the pandemic, including curfews and lockdowns.^17^

The editors of the special summer 2020 issue of *Policy and Society* state in their introduction that the articles presented reveal

how the national responses to the Covid-19 pandemic were shaped by the opportunity and capacity each government had to learn from previous pandemics and their capacity to operationalize and build political support for the standard portfolio of policy measures deployed to deal with the crisis.^18^

One article utilises historical and cultural perspectives to address Sweden's initial response and explain the countermeasures that were implemented. The author remarks that

Strategies that aim to alter social behavior must depart from the pre-existing patterns of behavior and the inclination among citizens to follow government guidelines. In some cultures, such guidelines have to be enforced with severe sanctions for those who ignore the guidelines whereas in other cultures the government can rely on using more subtle means.^19^

Sweden would fall into the latter category insofar as it has used nudges and recommendations to ensure social distancing. This relies upon the existence of a culture of trust between authorities and citizen.^20^

Yan and colleagues note similar findings in their comparative study involving Sweden, China, Japan, and France.^21^ Another comparative study involving Germany, France, and Sweden suggests that administrative tradition and culture may explain why countries have taken such different paths. The Swedish voluntary strategy was thus described as residing upon the culture of public administration, a high level of trust in public authorities, and trust in advanced heath care.^22^ Other research regarding the ‘relaxed’ Swedish Covid-19 strategy stated that the country's administrative tradition of dualism in respect to political institutions and administrative agencies, coupled with the strong decentralisation of national, regional, and local levels of administration, led to substantial difficulties with coordinating different administrative levels.^23^ Another study concluded that the structure of public administration and consensus-oriented public debate underlay the difficulties encountered in efforts to control the decentralised system and change the initial strategy.^24^ Kuhlmann and colleagues simply remarked that Sweden was the one country in their study that did not centralise control during the crisis, but they were unable to explain why.^25^

It is thus not always clear why given countries chose one type of policy measures and maintained one and same approach during the Covid-19 pandemic. Reider Staupe-Delgado^26^ reveals that most states changed their strategic posture several times, which may have been due to political debate, critique, or their specific epidemiological situation. It is important to remember that most states were forced to produce policy responses with limited information and knowledge, which may or may not have led to later changes in their approach. Countries also began vaccination programmes in spring 2021, which rendered new policies and approaches feasible.^27^ Sweden has instead maintained a relatively stable strategy throughout the pandemic, with its initial policy of voluntarism being amended quite late with certain legal restrictions on businesses. Could there be a theoretical explanation for why voluntarism comprised the basis of the Swedish strategy and for why Sweden maintained its initial strategy?

Although there is now an extensive body of research regarding Covid-19 and policy responses to the pandemic, no study to date has focused on why securitisation ‘failed’ in Sweden and on why Sweden did not introduce more draconic and sovereign measures as mortality rates grew rapidly. The present study reveals that there was little possibility for politicians and leading experts to craft strong responses without violating constitutional and procedural frameworks, which they were unwilling to do. Such unwillingness, which is of great interest during a serious security event, makes sense in the theoretical light of path-dependency and a logic of appropriateness.^28^ The present study focuses on the arguments put forward in support of the general approach that was adopted and the particular countermeasures implemented as it explores the actions undertaken by the Swedish government and public authorities.

## The pandemic as a security threat

Regional virus outbreaks and global pandemics constitute a recurrent security problem that breeds fear among populations, health practitioners, security experts, crisis managers, and politicians. Not only have global and national security agendas been adjusted to explicitly incorporate ‘health security’ as a vital dimension of security in relation to virus outbreaks,^29^ pandemics have also been described as one of the ‘most feared’ security threats today.^30^ The Johns Hopkins Coronavirus Research Center reported 28 million confirmed infections and over 900,000 deaths worldwide at the beginning of September 2020, with the figures rising to nearly 229 million confirmed cases and over 4.6 million deaths on September 20th, 2021.^31^ By any standard, we may conclude that global pandemics in general, and Covid-19 in particular, may justifiably be regarded as serious security threats that demand the active governing of communications, individuals, and populations, both globally and nationally.

### Beyond securitisation theory

Securitisation theory currently comprises one of the more significant attempts to identify and understand the dynamics of security issues, and it has generally worked well in respect to the broader security agenda that arose after the end of the Cold War. While the notion of security had previously been exclusively associated with the threat, use, and control of military force,^32^ it came to include a host of other issues, including terrorism, global warming, natural disasters, global pandemics, and a variety of public health problems.^33^ Securitisation theory is based upon the premise that security issues do not exist by themselves, but are rather a product of contextual meaning-making insofar as security agents frame, argue for, and potentially gain acceptance for their specific views regarding a given issue, which they present as of great urgency and with high stakes implications. Security is thereby defined as ultimately a matter of life and death – in short, ‘security is about survival’.^34^

Risks of annihilation, or even existential threats in specific contexts, would then purportedly justify ‘the use of extraordinary measures to handle them’,^35^ although both threats and countermeasures must be regarded as credible. Buzan et al. state that

[T]he exact definition and criteria for securitization is constituted by the intersubjective establishment of an existential threat with a saliency sufficient to have substantial political effects.^36^

The process of securitisation is completed when an acceptance of breaking established rules emerges. Buzan et al. thus claim that

‘security’ is the move that takes politics ‘beyond’ the established rules of the games and elevates the issue as a special kind of political problem or as above politics.^37^

In contrast, Michael Williams argues that speaking about security and threats activates such emotions as fear and a general sense of danger, and that this serves to legitimise the implementation of solutions and measures that violate normal political procedures.^38^ Uncertainty and a sense of urgency thus constitute the social mechanisms for audience acceptance of extraordinary and extra-political measures. But what constitutes failed securitisation? Can we have an issue that is: (1) both urgent and a question of life and death; (2) expressed as such by political leaders and experts; but (3) does not successfully solicit acceptance of extraordinary measures by the audience? If such was the case, then we would need to explain why extraordinary measures were not employed and securitisation theory apparently failed.

It is difficult, however, to distinguish between ‘failed securitisation’ in the empirical sense and a ‘failure of securitisation theory’ to account for weak responses to apparently serious security threats, such as the Covid-19 pandemic. Scholars have previously claimed in this regard that we need to disaggregate the process of securitisation if we wish to understand ‘failed’ securitisation.^39^

Mark Salter states that securitisation may fail in three specific ways that comprise ‘normal’, internal, or external failure. In situations of normal failure, a specific policy may fail in its implementation phase regardless of whether it reflects convincing speech acts.^40^ Securitisation would fail internally if the framing and/or connection between threat and countermeasures is not logical. External failure indicates that exceptional measures and the specific solution have been rejected as inadequate or inappropriate in respect to a specific security issue.^41^ Although Salter's work is useful for gaining an understanding of both failed securitisation and the process of securitisation, these three types of failure clearly remain within the context of speech acts, acceptance, and extraordinary countermeasures that break rules and conventions. Furthermore, the issue of success or failure does not question the ontological, theoretical, and philosophical underpinnings of the view that security by nature is extra-political.

Andrew Neal maintains that securitisation theory is ‘the archetypical expression of security as anti-politics’.^42^ Stated otherwise, it resides upon the conception that socially constructed security issues are, by definition and in practice, lifted from their context, attached to procedures of normal politics, and then placed into a context of executive decisions and exceptional and extraordinary measures that involve breaking established rules and violating constitutional rights and democratic procedures. We must note, however, that security and crisis management not only comprises a key aspect of what states do on a regular basis, it also is a central element of state organisation.

Analyses of security, including critical and normative discussions of this question, are largely preoccupied today with sovereign and executive powers and do not include embedded state theory.^43^ We nevertheless continue to conceive of security in the shadow of Thomas Hobbes's notion of the Leviathan, including his preoccupation with security as prior to politics and the joys of life. Neal terms this the ‘Hobbesian trap’.^44^ We should keep in mind, however, that states regularly engage in contingency planning, which is an essential concern of public authorities in general, not only specialised agencies. Anticipation of what may happen; adequate allocation of resources, personnel, and equipment; crisis and emergency planning and organisation; predefined tasks; responsibilities and regulations; instruction and training; and decision guidance are often in place prior to the emergence of any acute crisis.^45^ It is therefore not self-evident that every crisis – or even crises as such – triggers an exception to accepted rules and procedures, thereby invoking a state of exception, exceptional measures, and true sovereign and executive power. Scholars nevertheless continue to analyse such events as if they always invoked Schmittian uncertainty. In Schmitt's words,

The precise details of an emergency cannot be anticipated, nor can one spell out what may take place in such a case, especially when it is truly a matter of an extreme emergency and how it is to be eliminated.^46^

The idea that security management is inherently illiberal and undemocratic has in fact been a key principle in the work of critical security scholars. Fleur Johns remarks, for instance, that security and the notion of exceptionalism ‘rings liberal alarm bells’.^47^ Jef Huysmans observes in the same vein that

[O]ne of the key characteristics of the jargon of exception is its suppression of the political renditions of the societal. In doing so, it eliminates one of the constituting categories of modern democratic politics, hence producing an impoverished and ultimately illusory understanding of the political contestation and domination.^48^

Huysmans further argues that exceptionalism ‘erases the societal as a realm of multifaceted, historically structured political mediations and mobilizations’ because of the specific way in which it frames political problems and solutions.^49^ Security and crisis events thereby allegedly constitute moments in which executive powers, to varying degrees, come to take precedence over democratic institutions and principles.

In contrast to the rather strong assertion that security is (always) extra-political in character insofar as it introduces and operates within the political vacuum of exceptionalism, we may instead emphasise how organisational and institutional designs and structures tie governments and leading authorities to existing legal frameworks, norms, and procedures.^50^ Institutional design reflects the shared rules, norms, and belief systems that have been established and accepted as guidelines for the social and political behaviour of politicians. Moreover, it shapes the nature of decision-making, coordination, and information sharing processes even during periods of crisis. Crisis and security management does not take place on a blank slate, in spite of the fact that security threats raise the stakes and demand coordination as well as swift decision-making and implementation.^51^ Even when states encounter something as frightening as a rapidly spreading virus, existing structures and crisis management systems are likely to bind political actors to common rules and procedures and crisis management organisation.^52^

Political leaders, the state bureaucracy, and expert specialists – perhaps above all in situations of uncertainty – thus search for appropriate, accepted, and legitimate ways to act in accordance with existing rules and procedures.^53^ March and Olsen refer to this as the logic of appropriateness, stating that

to act appropriately is to proceed according to the institutionalized practices of a collectivity, based on mutual, and often tacit understandings of what is true, reasonable, natural, right, and good.^54^

Consequently, political actors do not necessarily seek power or future utility, but might instead adhere to existing formal and informal institutions and normative considerations so that they could be viewed as acting appropriately in respect to the public roles they held. We would thus expect them to not seize the opportunity to disrupt existing legal frameworks and procedures in order to be regarded as sovereign executive actors, even during serious security events.

Path dependency, potential disruptions, and alterations in policy responses must also be taken into consideration in respect to an event that unfolds over a longer period of time. James Mahoney discusses path dependency without disruptions as comprising *self-reinforcing sequences*, remarking that

an [institutional] pattern – once adopted – delivers increasing benefits with its continued adoption, and thus over time it becomes more and more difficult to transform the pattern or select previously available options, even if these alternative options would have been more ‘efficient’.^55^

In contrast, Ian Greener maintains that path dependency does not necessarily involve increasing returns insofar as a given policy, once implemented, tends to acquire momentum because of ideological or ideational lock-ins.^56^ Institutional or policy reproduction may also be based upon legitimation, such as when actors voluntarily opt for such reproduction because they regard a given institution as legitimate.^57^ We must also take into account the fact that all historical events give rise to possible breaches of the given path, such as when actors, in reactive sequences, reconsider previous choices. Path dependency may then be broken if actors find the current path to be illegitimate or substantially faulty.^58^

There are thus sound theoretical perspectives other than ‘failed’ securitisation from which to investigate how democratic states are limited by constitutional and institutional structures that strictly hinder central actors from breaking rules and procedures, even when they are struggling with a serious security issue.

## Methods and materials

The analysis of the Swedish case that this article presents focuses on the legal framework for and organisation of the Swedish security and crisis management system insofar as they form the basis for responding to any emerging security events. Of great interest is whether, when, and how the Swedish government and the responsible public agencies engaged in ‘securitisation moves’, including how the Covid-19 pandemic was understood and communicated to the wider public. How the problem was understood, along with the constitutional and organisational system for the overall management of security issues, provides the theoretical starting point for an analysis and explanation of the Swedish Covid-19 strategy.

An analysis concerned with the framing of security problems and the possible solutions thereby implied can fruitfully utilise Carol Bacchi's approach to policy analysis, which she terms ‘What's the Problem Represented to Be?’. This approach is based upon the premise that solutions to policy problems follow from both the implicit and explicit framing/representation of a given problem. This accords well with the basic premises of securitisation theory in that it focuses on speech acts and framings as key aspects of ‘producing and solving’ problems.^59^ However, the addition of factors associated with appropriate behaviour and bounded rationality, which suggest that policy formation is also shaped by organisational and institutional structures, generates a complex picture that makes it necessary to investigate the reasons put forward for specific policies and countermeasures.

The present study, which examines the period from when the first case of Covid-19 was identified in Sweden in January 2020 to September 2021, traces policies and steering techniques as they were developed and implemented as well as the later suspension of the countermeasures that had been employed. It is challenging to analyse a moving target – the pandemic remains ongoing, and policy responses are continually adjusted in accordance with the current situation and emerging knowledge of the virus, in both Sweden and all other countries. Our investigation addresses problem descriptions, framings, solutions, and implementations. The outcome, or dependent variable, is not the effectiveness of the Swedish Covid-19 strategy. It rather consists of specific policy instruments and their underlying motivations insofar as they serve to explain the controversial Swedish strategy, regardless of its effectiveness.

There is a copious amount of relevant material available in Sweden, which is somewhat uncommon for qualitative policy analysis. One reason for this is the fact that the Swedish political system is transparent, with all public documents being readily available on official websites. I have relied in this article primarily on public materials from the government, the parliament, and the Public Health Agency of Sweden (PHAS) (*Folkhälsomyndigheten*). The PHAS took a leading role as an expert authority soon after the appearance of Covid-19 in the country, and it published weekly reports on the situation and the actions taken. It also held almost daily press conferences, which are available on their designated YouTube channel.^60^ The Committee on the Constitution held a public hearing with Prime Minister Stefan Löfven on 26 April 2021, and this has also been included in the material analysed during revisions of the manuscript.^61^ My previous familiarity with the Swedish security and crisis management system has been valuable in that it has provided guidance regarding structures, standard procedures, and the most important actors.^62^ I employed qualitative content analysis in my investigation of documents and media presentations^63^ in order to extract important passages of theoretical relevance. Although I examined mainly written texts and spoken words, I also included certain empirical events, conflicts, and critiques in order to identify the political aspects of the strategies chosen. The material analysed has made it possible to present an unfolding chronological narrative that reveals the construction of the problem and the possible solutions available underlying security management of the Covid-19 pandemic in Sweden.

## The Swedish response to Covid-19

The first case of Covid-19 in Sweden was confirmed on 31 January 2020, in Jönköping,^64^ after a young woman had arrived in Göteborg from Wuhan. Internal spread within Swedish society was not confirmed until 6 March. The first weekly report by the PHAS, for the period 9–15 March, noted that most cases (63 per cent) could still be traced to travelling outside Sweden or to a close connection with persons returning from abroad, particularly those who had visited ski resorts in Italy and Austria. One thousand cases of Covid-19 infections were discovered that week, and there were clear indications of an emerging spread within the population. Sweden recorded its first fatal case on 11 March, which was the same day that the WHO announced that Covid-19 should be regarded as a global pandemic. The Swedish government and the responsible authorities became acutely aware of the seriousness of the situation quite early, in spite of the fact that there were only 200,000 cases identified in the world in mid-March. The global spread of the infection had been confirmed through the discovery of Covid-19 cases in 159 different countries.^65^

## The Swedish administrative state and the crisis management system

The PHAS normally bears the primary responsibility for managing transmittable diseases. Its national mandate is to work in a comprehensive manner to ensure good public health, above all to protect the population from communicable diseases and other health threats – the vision statement of the agency is ‘a public health that strengthens the positive development of society’.^66^ The PHAS played a decisive role as the leading expert authority in crafting the Swedish Covid-19 strategy, and the head of the agency and its key experts quickly become publicly known through daily press conferences. Its Chief Epidemiologist, Anders Tegnell, is now known throughout the world as the man behind the Swedish Covid-19 strategy. He held almost daily press conferences during the first three months of the pandemic and participated in national television and radio broadcasts and other media events several times a week. More importantly, Tegnell and the PHAS recommended measures that the government should take to combat Covid-19 throughout the period of time addressed by this article, including the first wave and the initial response.^67^

It may appear unusual that the government and the executive authorities did not play a more prominent role during this period. Rather than seize the moment and expand sovereign and executive powers, the government and Prime Minister Stefan Löfven continued to adhere to the strong Swedish dualism noted above, acknowledging the important role played by leading expert authorities. This dualism resides in large part upon the historical and constitutionally codified relationship between the government and public agencies. We should emphasise that public agencies enjoy a high degree of autonomy in respect to the government, even though the constitution stipulates that the covernment ‘controls and governs’ them. This is a result of Sweden's historical political development, in which democratic reforms began under a regime of strong monarchical rule. At that time, one way in which to ensure that public agencies followed the laws approved by the parliament (and the people), instead of being controlled by the executive power (the king), was to codify the independence of public authorities in the constitution. This accords with a robust Montesquieuan ideal regarding the separation and balance of powers (executive, legislative, and judicial) that is uncommon for parliamentary systems, and it significantly restricts the executive power of the government and the prime minister in respect to the bureaucracy.^68^ For example, not only are government ministers limited in their ability to govern and control the public agencies in their respective political sectors, they can be reported to the committee on the constitution for any attempt to exert direct control over agencies and specific assignments. Their direct involvement in the work of a public agency normally warrants a report of ‘ministerial rule’, which is unconstitutional in Sweden.^69^ There were no reports of ministerial rule during the initial phase of the pandemic, and the mix of formal and informal institutions appears to have remained intact.

Because of these strong historical institutions, it is the various departments within the government that conduct policymaking, including the organisation of public commissions and the drafting of bills to be submitted to parliament. In addition, public agencies are responsible for the implementation of laws once they have been approved by parliamentary vote. It is noteworthy that existing legal frameworks stipulate that ministerial departments and public agencies may not have even informal contacts with each other, although certain crisis situations may both require and make possible various informal meetings and a coordination of activities. Although it would have been interesting to know whether such informal meetings or briefings had taken place involving the government, the Secretariat for Crisis Management (discussed below), and the PHAS during the period we have investigated, it is difficult to obtain information in this regard. It is clear that the government implemented no policies during that period that deviated from the public statements of the PHAS, which serves to support the conclusion that the Swedish government followed the advice of the expert authority. Prime Minister Lövfen pointed out during the 26 April 2020, public hearing with the committee on the constitution that it was crucial to listen to expert authorities when crafting policies, but that final responsibility rested with the government in light of the governmental decision order. Löfven in fact argued that it would be odd to *not* listen to experts given the nature of the crisis.^70^

When the prime minister was pressured on the point of who was leading the country during the pandemic, he maintained that while he and the government were the leading actors, they *had not* been willing to govern through executive power or violate any established regulations. Following rules and procedures was key to managing the pandemic, and this often involved indirect governance by means of regulations, recommendations, and legislation. Not only did Löfven place no emphasis on the logic of securitisation during the public hearing, he projected a logic of appropriateness that involved following the formal and informal rules that pertain to the prime minister's role in Sweden and seeking the legitimate course of action.^71^ Institutional lock-ins are clearly difficult to disrupt even during crises.

An additional element in Sweden's administrative dualism is the tradition of strong local and regional bodies that have their own political leaders and administrations. The 21 Regional Boards in the country are the governing bodies in charge of hospitals and advanced healthcare, while the 290 municipalities are responsible for elderly care as well as basic healthcare. We must note that the regions and municipalities enjoy constitutional independence from the national government, much in the same way as the national public agencies.^72^ Sweden is thus a highly decentralised and embedded state, which makes it difficult for central authorities to seize control and govern during crises.

One might nonetheless regard the Covid-19 pandemic as a security issue of such severity that increased control and executive power would be needed to effectively coordinate all levels of the state, even with a highly decentralised form of government. However, Sweden has no legal framework or constitutionally codified conditions that apply to ‘national crises’ or ‘severe emergencies’ other than a formal state of exception, which the Form of Government explicitly indicates is possible only in the event of war.^73^ An earlier public investigation that comprised an overview of the legal framework for national crises observed that Sweden has only limited constitutional means other than certain references to ‘extraordinary events’ associated with war for circumventing normal procedures in times of emergency that would entail restrictions of current public law.

The regulations in Chapter 13 of the Form of Government (RF) increase the room for action on the part of the highest state bodies under ‘the extraordinary conditions caused by war or the threat of war' so that the measures demanded by the situation can be taken. However, there are no corresponding regulations that apply to civil crises, which must then be handled in accordance with the general rules that pertain to the form of government.^74^

Stated briefly, the Swedish security and crisis management system has been developed primarily for peacetime emergencies, and it accords to a great extent with the constitutional order and decentralised political system described above. This type of system, coupled with a long period of peace, has permitted Sweden to put a halt to war preparedness. This includes preparedness for national defence as well as large-scale national and international crises, although a return to total defence emerged in 2014 after the Russian annexation of Crimea.^75^

A closer look at the Swedish crisis management system, which is focused on societal security, reveals that it resides upon three official principles for non-military security measures:

1. The principle of responsibility: Whoever is responsible for operations under normal conditions should bear the equivalent responsibility during crisis situations.
2. The principle of similarity: The organisation of any function in crisis situations should remain as similar as possible to its normal status.
3. The principle of subsidiarity: Crisis and security challenges should be managed at the lowest possible level.

These principles were introduced at the turn of the current century in order to adapt Swedish security management to societal rather than solely military security. They were intended to create a flexible and robust system that would be able to meet security challenges at the local, regional, and national levels.^76^ It is noteworthy that the Swedish Civil Contingencies Agency (MSB), which was established in 2009 as the main public agency responsible for crisis management, has no operative functions. Its aim is instead to monitor civil protection, public safety, emergency management, and civil defence when no other authority bears responsibility. The MSB thus serves as an expert public agency that gathers knowledge in order to assist in coordinating activities and providing information to the public, but it has no mandate to govern other actors, public or private, at any level. The pandemic has made it evident that the principal responsibilities of the MSB, the PHAS, the National Board of Health and Welfare (NBHW),^77^ the regional boards in charge of health care, and the municipal agencies in charge of elderly care and other social welfare are limited to ensuring a substantial level of coordination. This highlights the difficulties in practice of implementing the three principles of the crisis management system.

Swedish security and crisis management had previously been criticised for being slow to react to a number of security events that occurred in the early 2000s. In response, the Swedish government took the decision to establish a Secretariat for Crisis Management in the Government Offices in order to help bridge the strict divisions between the government and public agencies. Its purpose was to facilitate coordination between these agencies and the government and among the agencies themselves. The secretariat was empowered to bring together relevant experts and administrators to support the prime minister and the state secretary to the minister for home affairs by developing, coordinating, and following up on crisis management in the government offices and monitoring the national crisis management system as a whole. However, the secretariat is only a support institution for the government – it has no operative functions and issues no decisions on its own authority.^78^

The Crisis Management Council, headed by the state secretary to the minister for home affairs, was established by the government in December 2008 as a forum for information sharing between agencies and the government offices.^79^ It meets twice yearly for contingency planning as well as during significant national crises, with the meetings attended by members of the council and representatives of the government offices. Overall security and crisis management is highly decentraliaed, and it requires careful coordination between different administrative levels and across policy sectors to function effectively. Such a system is difficult to control and govern since there are substantial obstacles to forceful executive action. We should note that there is no indication in the material analysed that the government or leading expert authorities sought to circumvent the existing rules and practices of the current crisis management system.

## Failed securitisation or sticking by the book?

To this point we have analysed the potential implications of the Swedish political system in general, which is characterised by decentralisation and strong administrative dualism. We have also examined the country's current security and crisis management system, which maintains decentralised responsibility and involves no type of central coordination for addressing large-scale national crises. We have noted little evidence of rule breaking, but have observed that rule following and the logic of appropriateness best describe how the government and leading public authorities have operated. One may argue that although Covid-19 was declared to be a global pandemic and Sweden recorded the internal spread of the virus at the beginning of March 2020, the government and the PHAS could not achieve effective central control and coordination simply because the ‘system’ would not permit it.

Nevertheless, evidence presented below indicates that both the government and the PHAS did in fact regard the pandemic as a serious security threat to Swedish society at large and, indirectly, to the state as well. For example, Prime Minister Stefan Löfven made a televised address to the nation on 22 March that was prompted by the exceptional situation. He stated that

Every person now has to mentally prepare for what awaits. We have a general spread of [Covid-19] in Sweden. Life, health, and jobs are threatened. More will become sick, more will be forced to say their final farewell to a loved one. The only way to cope with this is to face the crisis as a society in which everyone takes responsibility for themselves, for each other, and for our country.^80^

No Swedish prime minister had made such a public address since the Second World War.

In addition, the PHAS requested that the government formally declare that Covid-19 was a ‘threat to public health’, which would allow them to treat the virus as a public health issue. This new status, which went into effect on 1 February^81^ required that both suspected and confirmed individual cases be reported to the authorities so that regional contagious disease specialists could issue recommendations and regulations aimed at containing clusters and local outbreaks of Covid-19. This also made it possible to quarantine individuals and enforce their isolation, although this option has never been exercised during the entire pandemic.^82^

The State Secretary to the Minister for Home Affairs, Elisabeth Backteman, called for a meeting of the Crisis Management Council on 2 March 2020, in order to ensure that thorough preparations were underway for the emerging pandemic. A further move towards political action took place on 6 March, when the PHAS held its initial meeting with the newly installed National Pandemic Group (NPG). This group was created in December 2019 following a prior overview conducted by the PHAS that had been unrelated to the Covid-19 pandemic. The NPG includes representatives from the PHAS, the MSB, the NBHW, the Swedish Association of Local Authorities and Regions, and other public actors involved in the national health care system.^83^

The PHAS held a press conference on 13 March, one week after the NPG meeting, at which the Director General admitted that precautionary measures had not been effective, and that Sweden must face the fact that ‘the pandemic is upon us’. Sweden had only 755 known Covid-19 cases on that date, but the majority could not be traced to visits abroad. The PHAS stated that the situation would get ‘much worse’, and that the regions and municipalities needed to be prepared for a great number of infections, including patients in need of intensive care. There was a strong concern that a steep climb in the rate of new infections would risk overburdening the national health care system, and that the resources available for intensive medical care might not cover future needs.^84^

Sweden recorded its second Covid-19 death on 14 March, the day after the PHAS press conference. This was taken as an indication that the disease was beginning to unfold into a national crisis that would require specific attention, and that the national health care system was indeed at risk. Measures were then taken to redirect national health care to focus on Covid-19 patients, including the expansion of intensive care facilities in order to manage a deteriorating situation. Even if certain patients had to be transferred between regions, there are no reported cases in which patients did not have access to intensive care.^85^ It is thus evident that Covid-19 was quickly considered to be a serious security threat, but that all actions taken closely followed established rules and procedures.

## Crafting countermeasures

The PHAS's primary aim was to ‘fight Covid-19 and minimize the death rate and other negative consequences for individuals and society’.^86^ The PHAS maintained that both medical and non-medical measures were necessary in order to attain these goals, together with a significant level of public communication directed as making all actors aware of what they could do to help stop the spread of the virus. They hoped to flatten the curve in this way and thereby not overwhelm the available resources of the national health care system.^87^ The PHAS and the government also highlighted two issues that substantially shaped the Swedish Covid-19 strategy. First, specific measures needed to be implemented at precisely the proper time in order to curtail the internal spread of Covid-19 within society.^88^ Second, such measures needed to be tempered in order to be accepted by the wider public and thus be sustainable over the long term at both the individual and societal levels.

During March 2020, the government issued a broad set of recommendations and restrictions that were in strict accord with advice from the PHAS. This included recommendations to avoid visiting healthcare institutions and elderly care homes (later adjusted to a prohibition); remain at home if you have any symptoms; work from home; avoid contact with elderly persons; avoid travelling both domestically and abroad; restrict the number of people attending public gatherings to no more than five hundred (later adjusted to fifty); and suspend campus-based education in secondary schools and universities. Restaurants and cafés were not closed, but were required to secure social distancing between clients (restaurants have been inspected and closed if over-crowded). This was amended on 19 November with a ban on alcohol consumption in restaurants and cafés after 10.00 pm, which was extended on 24 December to a ban on alcohol consumption after 8.00 pm, and a limit on seating of four people per table. This list indicates the range of regulations and recommendations issued early in the pandemic that have remained in effect for lengthy periods of time, in some cases for close to a year, with some having been amended with stricter measures. The legal basis for such regulations, as well as the regulations themselves, are discussed in greater detail in the next section.

The government adopted the position in April 2020 that it was unable to act in the manner necessary when new clusters of Covid-19 outbreaks were identified because of constitutional constraints on executive power, particularly if one acted in strict accord with the Swedish crisis management system. After surveying the political situation in the parliament and discussing the matter with the Council on Legislation, which is an advisory board for the government and the parliament, the government presented a bill for a time-limited Pandemic Law that would provide it with the power to issue regulations for dealing with Covid-19. While such regulations are not laws in a strict sense, they have a similar status and effect in that they oversee the actions of public authorities.

The parliament granted the government the additional power requested by means of a time-limited amendment to the Communicable Diseases Act.^89^ This referred solely to Covid-19 and sanctioned issuing regulations in order to resolve specific situations and redistribute medicines and medical equipment between both public hospitals and private health care providers. Limited executive power that strictly concerned government control over public authorities was thus made possible only after formal approval by the parliament. This amendment was never used, however, and it was in effect only between 18 April and 1 July.^90^ When infection rates exploded during the fall of 2020, the government once again found it had no support or legal ability to govern beyond recommendations. Consequently, a new Pandemic Law was prepared during the autumn of 2020, which was adopted by parliament after extensive review on 8 January 2021, coming into effect on 10 January. This law was primarily directed to controlling and restricting the operations of various types of businesses, including restaurants, exercise facilities, malls, sports facilities, libraries, campsites, museums, zoos, shopping malls, shops, service facilities such as hairdressing salons, party rooms, and public transport. Limits were also set for how many people could attend private gatherings. Regional boards and authorities were assigned supervisory responsibility regarding the corresponding measures. This new law provided the government and public authorities with greater leverage in controlling public spaces and private businesses, and made it possible to issue fines and close businesses that violated the applicable regulations.^91^

These developments clearly indicate that the underlying idea in securitisation theory concerning breaking existing rules is not supported in the Swedish case, even during serious crises. It is rather the logic of appropriateness, or *rule following*, that provides an explanation for how the government may take action, which at times proceeds slowly. The executive power of the government has consequently remained at a moderate level, regardless of the serious security threat posed by the pandemic. Although political action was taken, it remained within constitutional boundaries. The lack of resolute action, and the fact that it took close to a year to adopt a pandemic law, have been heavily criticised by the media and the opposition.

## Regulations, recommendations, and individual responsibility

Our analysis has revealed that although Covid-19 was viewed as a serious security threat, all the actions taken, including the regulations and recommendations implemented, accorded with existing constitutional and organisational procedures and practices. This appears to have prevented a securitisation process that culminated in rule breaking, with the government and other key actors appearing eager to not violate existing norms. However, the fact that all countermeasures had to be based upon existing laws left little room for placing restrictions upon businesses and the movements of individuals, and it took close to a year to legislate such capabilities. The government and the responsible public authorities consequently had to rely upon governing strategies other than lockdowns or curfews to ensure social distancing among the population. There were thus few options other than employing indirect governing strategies, that is, policies that relied upon individual responsibility and utilised such instruments of governing as information, recommendations, and appeals to modify individual behaviour.

The negative health effects of the pandemic involved not only the risk of being infected, but also the negative effects upon individuals of the countermeasures themselves. This has generated substantial public debate concerning the need to weigh the negative consequences and the various groups affected. For example, day care centres and elementary schools had to remain open, even if Sweden implemented a partial school closure in light of the argument put forward by the PHAS that a complete school closure would have strong negative consequences for society. Not only would many people who performed key societal tasks have to remain home to care for young children, there would also have been severe disruptions in the well-being of those children who were dependent upon schools as ‘safe havens’. Secondary schools, universities, and adult education centres, including secondary school completion programmes, were closed for in-house attendance between 18 March and 1 July, the beginning of the summer break.^92^ Both the PHAS and the government believed that adults and older pupils were capable of taking responsibility for their education even if it was moved to online platforms. The right to education and the School Law have continued to serve as the guiding principles for how to manage schools during the pandemic, even if adjustments have been made at all levels in accordance with the general recommendations concerning social distancing and public gatherings. Large university lectures have proceeded online, with only smaller seminars being conducted on campus. In addition, individual schools may be closed in the event of a local Covid-19 outbreak.^93^

The Swedish government issued a ban on all public gatherings and events of more than fifty people on 27 March 2020. This ban was amended on 24 November to stipulate a maximum of eight people, which is a severe restriction that infringes upon basic democratic rights since it also pertains to public demonstrations. The aim of this temporary legislation was to prevent social situations in which large numbers of people from different parts of the country would come together and then return home, potentially leading to a large-scale spread of the Covid-19 virus. Such situations included demonstrations, seminars, religious events, theater performances, concerts, sport events, and fairs. This was formalised as an amendment to the Public Order Act,^94^ which specifies that the government may restrict demonstrations and public gatherings in order to suppress the pandemic, but that any restrictions on the constitutionally protected right for public gatherings must be ‘proportional and reasonable’.^95^^,^^96^ In order to protect the elderly, the government issued a regulation prohibiting social visits to elderly care homes beginning 1 April 2020. This temporary regulation,^97^ which was aimed at preventing the spread of Covid-19 to vulnerable groups, was lifted on 1 October 2020. It had been formalised as an amendment to the general Social Law (*Sociallagen*) that makes it possible for the government to issue prohibitions necessary for protecting the lives, security, and well-being of individuals who are in ‘public care’.^98^^,^^99^

These government-issued regulations accord with and reside upon existing laws, and the amendments in question do not violate or exceed existing regulations and constitutional rights. The government has not employed executive power to enforce any countermeasures that do not accord with the views of experts or the political opposition. Furthermore, since the government has only limited powers to issue legal restrictions, it has had to rely upon recommendations and advice to the general public in accordance with the Communicable Diseases Act.^100^ This law emphasises that regions and municipalities are responsible for being prepared and equipped to deal with outbreaks of transmittable diseases. It also states that individual citizens should ‘be alert and take precautions to actively contribute to stopping the spread of transmittable diseases’. It further stipulates that ‘any restrictions on individual liberty may only be invoked if no other options are available’.^101^ While recommendations themselves are not binding, the PHAS remarks that ‘a recommendation is based on all the knowledge available on a particular subject, which means that it is a good idea to follow any recommendation issued by an authority’.^102^

As noted above, personal responsibility is a key dimension of the Swedish Covid-19 strategy.^103^ National informational campaigns, communications, and press conferences by the government and the PHAS have continuously repeated the message that accepting one's personal responsibility constitutes an act of solidarity with others, particularly vulnerable groups. This expresses the need for individuals to be steadfast and careful, for both their own sake and that of others.^104^^,^^105^ For example, Prime Minister Löfven stated in an 22 April 2020 government press conference that

It is not yet time to relax, we are still in the middle of this crisis … Recommendations from public authorities are … based on existing knowledge, and they aim to secure your personal well-being as well as the well-being of society at large … We can only do this together.^106^

The more general and long-term recommendations that have been put forward for dealing with the pandemic include the following:

- Stay home even if you have only mild symptoms or a light cold.
- If you have Covid-19 symptoms that last longer than 24 hours, contact local health care providers to be tested for Covid-19.
- Wash your hands frequently with soap and water for at least twenty seconds.
- Keep an arm's length away from others both indoors and outdoors.
- Avoid public transit if possible.
- Maintain distance from others on buses, subways, trams, and other public transit.
- Avoid participating in private social events such as parties, funerals, baptisms, and weddings.
- Keep a distance from others at sports facilities, swimming pools, and gyms, and avoid changing clothes in public dressing rooms.
- If you are seventy or older, avoid places where people gather and limit your physical contacts at all times (PHAS).^107^^,^^108^

The role of personal responsibility in the Swedish approach to Covid-19 is perhaps the most controversial issue in comparison with other countries, since it places the responsibility for social distancing on the individual, not on state authorities. Prime Minister Löfven stated in a press conference on 29 May 2020, that following recommendations for social distancing should be regarded as an ‘act of solidarity’ and a ‘moral obligation’ towards society in the absence of strict laws.^109^

We should note that there has been substantial national and international debate regarding the voluntary aspects of the Swedish Covid-19 strategy. In May 2020, a group of 22 scientists published a strong statement in one of the largest Swedish newspapers that indicates the level of ongoing political debate in Sweden over the strategy and its effectiveness. They heavily criticised the PHAS, the Swedish strategy as such, and the main figure behind that strategy, Chief Epidemiologist Anders Tegnell,^110^ their main point being that the strategy must be regarded as a failure insofar as the death toll in Sweden had come to exceed those of the other Nordic countries. In addition, the opposition parties in parliament demanded the appointment of an independent Corona Commission that would examine the Swedish Covid-19 strategy and the government's overall security and crisis management during the pandemic. Although the government sought to wait until after the crisis was over to appoint the commission, it was established on 30 June 2020, after opposition political parties exerted pressure that it be done immediately.

Such events reveal that there has been an ongoing scrutiny of executive power during the Covid-19 crisis as well as a more general political debate in which various experts have sharply criticised the official strategy for dealing with the pandemic. This indicates a rather high level of politic activity and opposition during the pandemic, with no ‘rally round the flag’ atmosphere. Furthermore, the international community and the international media have criticised the Swedish Covid-19 strategy for overlooking individual deaths in order to protect the economy, or even for allegedly conducting a socio-medical experiment aimed at herd immunity. The prime minister categorically dismissed such supposedly hidden motives during the 26 April 2021 public hearing.^111^

The government announced in a press conference on 27 May 2021 that Sweden would follow a five-step plan for easing restrictions and returning to ‘normality’ beginning 1 June. Sweden had begun its vaccination programme in January 2021, focusing on the elderly (over seventy years of age) and people with underlying health conditions.^112^ This meant that the pressure on public health facilities would slowly but surely ease, and most hospitals were able to return to normal status from heightened alert in February. The fourth step, which involved lifting the recommendation to work from home as well as restrictions on public gatherings, restaurants, nightclubs, and private gatherings, was announced for 29 September. The final step, not yet announced, will lift all remaining restrictions.^113^

Our analysis has revealed that rule following, the logic of appropriateness, and a reliance upon various legal means for regulating circulation in society have apparently constituted the underlying rationale for the Swedish Covid-19 strategy. New restrictions were nonetheless issued on the basis of the 8 January 2021 Pandemic Law. Although Swedish authorities in 2020 had quickly identified the pandemic as a serious security issue that would greatly affect both society and the state, they followed existing political protocols that were oriented towards societal security and small-scale incidents. It is currently unclear whether any legal changes will be instituted in light of the Covid-19 pandemic and the way in which it was managed. It is close to certain that both the government and parliament will wait for the report of the Corona Commission before initiating any substantial changes in the crisis management system or the constitution.

## Conclusion

Insofar as securitisation theory maintains that successful securitisation moves enable political leaders to break existing rules and procedures to avert an urgent security problem, one may say that the Swedish prime minister and the government ‘failed’ in their response to the Covid-19 pandemic. However, the material I have presented here clearly indicates that the overall approach employed in Sweden, including the countermeasures taken, was developed in accordance with the existing organisational, institutional, and constitutional rules and procedures, not securitisation theory. This entailed that a substantial period of time was needed to get most countermeasures in place. The material also reveals that political leaders and experts were well aware both of the seriousness of the unfolding global pandemic, and of public support and demands for decisive action, including various forms of lockdowns, curfews, and the closing of schools and businesses. Furthermore, although a review of the various countermeasures implemented indicates that at least some are quite restrictive, the image of a relaxed response lingers, perhaps because of the frequently repeated need for individual responsibility and voluntary compliance with general recommendations in order to stop the spread of the virus.

How can we then explain the formation of the Swedish Covid-19 strategy – or rather the patchwork of countermeasures that at times has generated unclear rules and signals as well as substantial friction and political debate? I would argue that the Swedish strategy was not simply a result of ‘failed securitisation’, but is rather associated with bounded rationality and a logic based on adhering to the organisational, institutional, and constitutional restrictions upon executive and sovereign power that characterises the Swedish political system in general and the current crisis management system in particular. Although this might be regarded as a highly unorthodox manner of coping with security issues that may not be completely successful in minimising mortality rates, previous studies have noted that individual responsibility for coping with crises and security has often been a part of Swedish security management.^114^ Whereas previous studies have focused on Sweden's administrative and cultural orientations, including a high level of trust in public institutions, the present study reveals that the Swedish Covid-19 strategy has emerged primarily from a combination of the administrative system, the present crisis management system, which focuses on societal security and small events, and a logic of appropriateness associated with acting correctly within existing institutional, constitutional, and organisation boundaries.

I would like to emphasise that the process of securitisation as described by securitisation theory has not taken place in Sweden during the pandemic insofar as the executive power of the government has remained substantially limited and existing rules or procedures have not been broken. The theoretical implications of the present study are thus of great significance for the work of security scholars. Andrew Neal^115^ remarks that we often fall into Hobbesian traps by assuming that security management (almost) always entails exceptions to existing rules and procedures, and we take it to be a case of failed securitisation if it does not. The present study reveals that *rule following*, not *rule breaking*, characterises Covid-19 management in Sweden. Although our investigation consists of a single case study that focuses on understanding and explaining the Swedish Covid-19 strategy, I maintain that our preliminary theoretical insights constitute an important contribution regarding how we should conceive of contemporary security management in democratic states. Sweden may be an exceptional case in that the overall political system is characterised by weak executive power. However, the Swedish approach to crisis management illustrates that securitisation theory overly emphasises rule breaking and exceptionalism, thereby blinding us to the importance and effectiveness of rule following and democratic-minded leadership in responding to serious security threats.

## Footnotes

1 Christian Enemark, ‘Is pandemic flu a security threat?’, *Survival*, 51:1 (2009), pp. 191–214, available at: {https://doi.org/10.1080/00396330902749798}; Simon Rushton and Jeremy Youde (eds), *Routledge Handbook of Global Health Security* (London, UK: Routledge, 2014); Rebecca Katz and Julie Fischer, ‘The revised international health regulations: A framework for global pandemic response’, *Global Health Governance*, 3:2 (2010), pp. 1–18.

2 Christian Enemark, ‘Ebola, disease-control, and the Security Council: From securitization to securing circulation’, *Journal of Global Security Studies*, 2:2 (2017), pp. 137–49, available at: {https://doi.org/10.1093/jogss/ogw030}.

3 Clare Wenham, Julia Smith, and Rosemary Morgan, ‘COVID-19: The gendered impacts of the outbreak’, *The Lancet*, 395:10227 (2020), pp. 846–48.

4 Barry Buzan, Ole Wæver, and Jaap de Wilde, *Security: A New Framework for Analysis* (Boulder, CO: Lynne Rienner Publishers, 1998).

5 Tom Christensen and Per Lægreid, ‘Balancing governance capacity and legitimacy: How the Norwegian government handled the COVID-19 crisis as a high performer’, *Public Administration Review*, 80:5 (2020), pp. 774–9, available at: {https://doi.org/10.1111/puar.13241}; Tom Christensen and Per Lægreid, ‘The coronavirus crisis: Crisis communication, meaning-making, and reputation management’, *International Public Management Journal*, 23:5 (2020), pp. 713–39, available at: {https://doi.org/10.1080/10967494.2020.1812455}.

6 Andrew Neal, *Security As Politics* (Edinburgh, UK: Edinburgh University Press, 2019), p. 6.

7 See: {https://www.bbc.com/news/world-europe-52395866}.

8 See: {https://www.nytimes.com/2020/07/07/business/sweden-economy-coronavirus.html}.

9 See: {https://www.abc.net.au/news/2020-08-20/swedens-herd-immunity-strategy-for-coronavirus-Covid-19/12570918}.

10 See: {https://edition.cnn.com/world/live-news/coronavirus-pandemic-08-31-20-intl/h_7ef6817282c1f566c8165b24dcb0a350}.

11 See: {https://www.who.int/emergencies/diseases/novel-coronavirus-2019/interactive-timeline}.

12 See: {https://www.who.int/news-room/detail/27-04-2020-who-timeline---Covid-19}.

13 In epidemiology, the idea of slowing the spread of a virus so that fewer people need to seek treatment at any given time is known as ‘flattening the curve’. See: {https://www.livescience.com/coronavirus-flatten-the-curve.html}.

14 Dimiter Toshkov, Brendan Carroll, and Kutsal Yesilkagit, ‘Government capacity, societal trust or party preferences: What accounts for the variety of national policy responses to the COVID-19 pandemic in Europe?’, *Journal of European Public Policy* (2021), available at: {https://doi.org/10.1080/13501763.2021.1928270}.

15 Hasan Muhammad Baniamin, Mizanur Rahman, and Mohammad Tareq Hasan, ‘The COVID-19 pandemic: Why are some countries coping more successfully than others?’, *Asia Pacific Journal of Public Administration*, 42:3 (2020), pp. 153–69, available at: {https://doi.org/10.1080/23276665.2020.1784769}.

16 Jae M. Moon, ‘Fighting against COVID-19 with agility, transparency, and participation: Wicked policy problems and new governance challenges’, *Public Administration Review*, 80:4 (2020), pp. 651–6, available at: {https://doi.org/10.1111/puar.13214}.

17 Christensen and Lægreid, ‘Balancing governance capacity and legitimacy’; Christensen and Lægreid, ‘The coronavirus crisis’.

18 G. Capano, M. H. Simon, D. S. L. Jarvis, M. Ramesh, and N. Goyal, ‘Mobilizing policy (in)capacity to fight COVID-19: Understanding variations in state responses’, *Policy and Society*, 39:3 (2020), pp. 285–308, available at: {https://doi.org/10.1080/14494035.2020.1787628}.

19 Jon Pierre, ‘Nudges against pandemics: Sweden's COVID-19 containment strategy in perspective’, *Policy and Society*, 39:3 (2020), pp. 478–93 (p. 491), available at: {https://doi.org/10.1080/14494035.2020.1783787}.

20 Pierre, ‘Nudges against pandemics’.

21 B. Yan, X. Zhang, L. Wu, H. Zhu, and B. Chen, ‘Why do countries respond differently to COVID-19? A comparative study of Sweden, China, France, and Japan’, *The American Review of Public Administration*, 50:6–7 (2020), pp. 762–9.

22 S. Kuhlmann, M. Hellstrøm, U. Ramberg, and R. Reiter, ‘Tracing divergence in crisis governance: Responses to the COVID-19 pandemic in France, Germany and Sweden compared’, *International Review of Administrative Sciences*, 87:3 (2021), pp. 556–75, available at: {https://doi.org/10.1177/0020852320979359}.

23 E. Petridou, ‘Politics and administration in times of crisis: Explaining the Swedish response to the COVID-19 crisis’, *European Policy Analysis*, 6 (2020), pp. 147–58, available at: {https://doi.org/10.1002/epa2.1095}; George Warren, Ragnar Lofstedt, and Jamie K. Wardman, ‘COVID-19: The winter lockdown strategy in five European nations’, *Journal of Risk Research*, 24:3–4 (2021), pp. 267–93, available at: {https://doi.org/10.1080/13669877.2021.1891802}; Gretchel Vogel, ‘“It's been so, so surreal”: Critics of Sweden's lax pandemic policies face fierce backlash’, *Science* (6 October 2020), available at: {https://doi.org/10.1126/science.abf1247}.

24 Staffan Andersson and Nicholas Aylott, ‘Sweden and Coronavirus: Unexceptional exceptionalism’, *Social Sciences*, 9:12 (2020), p. 232, available at: {https://doi.org/10.3390/socsci9120232}.

25 Ibid.

26 Reider Staupe-Delgado, ‘Life in anticipation of the COVID-19 pandemic “peak”: Reflecting on “strategies” for and variations in attempts at “flattening the curve” and managing the crisis’, *Journal of Extreme Events*, 8 (2021), available at: {https://doi.org/10.1142/S2345737621500160}.

27 Ibid., p. 18.

28 J. G. March and J. P. Olsen, ‘The logic of appropriateness’, in Robert E. Goodin (ed.), *The Oxford Handbook of Political Science* (Oxford, UK: Oxford University Press 2009), pp. 479–97, available at: {https://doi.org/10.1093/oxfordhb/9780199548453.003.0034}; James Mahoney, ‘Path dependence in historical sociology’, *Theory and Society*, 29:4 (2000), pp. 507–48, available at: {https://doi.org/10.1023/A:1007113830879}.

29 Stefan Elbe, Anne Roemer-Mahler, and Christopher Long, ‘Securing circulation pharmaceutically: Antiviral stockpiling and pandemic preparedness in the European Union’, *Security Dialogue*, 45:5 (2014), pp. 440–57 (p. 441), available at: {https://doi.org/10.1177/0967010614530072}; Adam Kamradt-Scott and Colin McInnes, ‘The securitisation of pandemic influenza: Framing, security and public policy’, *Global Public Health*, 7:Sup 2 (2012), pp. 95–110, available at: {https://doi.org/10.1080/17441692.2012.725752}; Katz and Fischer, ‘The revised international health regulations’.

30 See: {https://www.who.int/news-room/detail/27-04-2020-who-timeline---Covid-19}.

31 See: {https://coronavirus.jhu.edu/map.html}.

32 Stephen M. Walt, ‘The renaissance of security studies’, *International Studies Quarterly*, 35:2 (1991), pp. 211–39, available at: {https://doi.org/10.2307/2600471}.

33 Jef Huysmans, ‘What do you mean?: From concept to thick signifier’, *European Journal of International Relations*, 4:2 (1998), pp. 226–55, available at: {https://doi.org/10.1177/1354066198004002004}.

34 Buzan, Wæver, and de Wilde, *Security*, p. 28

35
Ibid

36 Ibid., p. 25

37 Ibid., p. 23.

38 Michael C. Williams, ‘Securitization and the liberalism of fear’, *Security Dialogue*, 42:4–5 (2011), pp. 453–63, available at: {https://doi.org/10.1177/0967010611418717}.

39 Paul Roe, ‘Actor, audience(s) and emergency measures: Securitization and the UK's decision to invade Iraq’, *Security Dialogue*, 39:6 (2008), pp. 615–35, available at: {https://doi.org/10.1177/0967010608098212}; Mark Salter, ‘When securitization fails: The hard case of counter-terrorism programs’, in Thierry Balzacq (ed.), *Securitization Theory: How Security Problems Emerge and Dissolve* (Abingdon, UK: Taylor and Francis, 2010), pp. 116–31 (p. 122).

40 Salter, ‘When securitization fails’, pp. 122–4.

41 Ibid., p. 125.

42 Neal, *Security As Politics*, p. 7.

43 Bengt Jacobsson, Jon Pierre, and Göran Sundström, *Governing the Embedded State: The Organizational Dimension of Governance* (Oxford, UK: Oxford University Press, 2015).

44 Neal, *Security As Politics*, p. 6.

45 K. Eriksson and A. McConnell, ‘Contingency planning for crisis management: Recipe for success or political fantasy?’, *Policy and Society*, 30:2 (2011), pp. 89–99, available at: {https://doi.org/10.1016/j.polsoc.2011.03.004}.

46 Carl Schmitt, *Political Theology: Four Chapters on the Concept of Sovereignty* (Chicago, IL: University of Chicago Press, 2005), p. 7.

47 Fleur Johns, ‘Guantánamo Bay and the annihilation of the exception’, *European Journal of International Law*, 16:4 (2005), pp. 613–35 (p. 620), available at: {https://doi.org/10.1093/ejil/chi135}.

48 Jef Huysmans, ‘The jargon of exception: On Schmitt, Agamben and the absence of political society’, *International Political Sociology*, 2:2 (2008), pp. 165–83 (p. 165), available at: {https://doi.org/10.1111/j.1749-5687.2008.00042.x}.

49 Ibid., p. 180.

50 Jacobsson, Pierre, and Sundström, *Governing the Embedded State*.

51 Arjen Boin, Paul ‘t Hart, Eric Stern, and Bengt Sandelius (eds), *The Politics of Crisis Management: Public Leadership under Pressure* (New York, NY: Cambridge University Press, 2005); L. K. Comfort, A. Boin, and C. C. Demchak (eds), *Designing Resilience: Preparing for Extreme Events* (Pittsburgh, PA: University of Pittsburgh Press, 2010).

52 R. M. Burton, B. Obel, and D. D. Håkonsson, *Organizational Design: A Step-by-Step Approach* (Cambridge, UK: Cambridge University Press, 2015).

53 P. J. DiMaggio and W. W. Powell, ‘The Iron Cage revisited: Institutional Isomorphism and collective rationality in organizational fields’, *American Sociological Review*, 48:2 (1983), pp. 147–60, available at: {https://doi.org/10.2307/2095101}.

54 March and Olsen, ‘The logic of appropriateness’.

55 Mahoney, ‘Path dependence in historical sociology’, p. 508.

56 Ian Greener, ‘The potential of path dependence in political studies’, *Politics*, 25:1 (2005), pp. 62–72, available at: {https://doi.org/10.1111/j.1467-9256.2005.00230.x}.

57 Mahoney, ‘Path dependence in historical sociology’.

58 Ibid.

59 Carol Bacchi and Susan Goodwin, *Poststructural Policy Analysis: A Guide to Practice* (New York, NY: Springer, 2016), pp. 28–9.

60 The PHAS held 157 press conferences by April 2021. See: {https://www.youtube.com/playlist?list=PLLqBo3UjMccAyAkJ9uiJkQpPjDYUoWlHp}.

61 See: {https://www.riksdagen.se/sv/sa-funkar-riksdagen/riksdagens-uppgifter/kontrollerar-regeringen/ku-utfragningar-om-granskningsarenden-2021/}.

62 Larsson (2018); Oscar L. Larsson and Sara Sjöqvist, ‘Managing national food security in the Global North: Is collaborative governance a possible route forward?’, *Risks Hazards Crisis Public Policy* (2021), pp. 1– 26, available at: {https://doi.org/10.1002/rhc3.12231}.

63 Kristina Boréus and Göran Bergström, *Analyzing Text and Discourse: Eight Approaches for the Social Sciences* (London, UK: SAGE, 2017), p. 7.

64 See: {https://www.folkhalsomyndigheten.se/nyheter-och-press/nyhetsarkiv/2020/januari/bekraftat-fall-av-nytt-coronavirus-i-sverige/}.

65 See: {https://www.folkhalsomyndigheten.se/globalassets/statistik-uppfoljning/smittsamma-sjukdomar/veckorapporter-covid-19/2020/covid-19-veckorapport-vecka-11-2020.pdf}.

66 See: {https://www.folkhalsomyndigheten.se/the-public-health-agency-of-sweden/}.

67 See the 26 April 2021 public hearing held by the committee on the constitution, available at: {https://www.riksdagen.se/sv/sa-funkar-riksdagen/riksdagens-uppgifter/kontrollerar-regeringen/ku-utfragningar-om-granskningsarenden-2021/}.

68 Jon Pierre, ‘Legitimacy, institutional change, and the politics of public administration in Sweden’, *International Political Science Review*, 14:4 (1993), pp. 387–401; Jacobsson, Pierre, and Sundström, *Governing the Embedded State*.

69 See: {https://www.riksdagen.se/sv/utskotten-eu-namnden/konstitutionsutskottet/}.

70 See: {https://www.youtube.com/watch?v=ZjO9QyNObkE} minute 13:35.

71 Ibid., minute 13:54.

72 Gun-Britt Trydegård and Mats Thorslund, ‘One uniform welfare state or a multitude of welfare municipalities? The evolution of local variation in Swedish elder care’, *Social Policy and Administration*, 44:4 (2010), pp. 495–511, available at: {https://doi.org/10.1111/j.1467-9515.2010.00725.x}.

73 SOU, *Krisberedskapen i grundlagen: Översyn och internationell utblick* (Stockholm: Fritzes Offentliga Publikationer, 2008), p. 61. Also see: {https://www.youtube.com/watch?v=ZjO9QyNObkE}.

74 SOU, *Krisberedskapen i grundlagen*, p. 61.

75 On national food security, see Oscar L. Larsson, ‘The connections between crisis and war preparedness in Sweden’, *Security Dialogue*, 52:4 (2021), pp. 306–24, available at: {https://doi:10.1177/0967010620936849}.

76 SOU, *Säkerhet i en ny tid: betankande* (Stockholm: Fritzes Offentliga Publikationer), pp. 14, 15–30.

77 See: {https://www.socialstyrelsen.se/en/about-us/emergency-preparedness/}.

78 See: {https://www.government.se/government-policy/emergency-preparedness/crisis-management-in-the-government-offices/}.

79 Ibid.

80 See: {https://www.regeringen.se/tal/2020/03/statsministerns-tal-till-nationen-den-22-mars-2020/}.

81 See: {https://www.hd.se/2020-02-01/regeringen-ger-besked-om-coronaviruset}.

82 See: {https://www.folkhalsomyndigheten.se/globalassets/statistik-uppfoljning/smittsamma-sjukdomar/veckorapporter-covid-19/2020/overvakningssystem-for-covid-19-v1.pdf}.

83 See: {https://www.folkhalsomyndigheten.se/smittskydd-beredskap/krisberedskap/pandemiberedskap/}.

84 See: {https://www.youtube.com/watch?v=5MFp_Olwu5E} minute 3:25.

85 See: {https://www.sll.se/verksamhet/halsa-och-vard/nyheter-halsa-och-vard/2020/06/alvsjo-sjukhus-avvecklas/}.

86 See: {https://www.folkhalsomyndigheten.se/smittskydd-beredskap/utbrott/aktuella-utbrott/covid-19/folkhalsomyndighetens-arbete-med-covid-19/}.

87 Ibid.

88 See: {https://www.regeringen.se/regeringens-politik/regeringens-arbete-med-anledning-av-nya-coronaviruset/strategi-med-anledning-av-det-nya-coronaviruset/}.

89 Smittskyddslagen (2004), p. 168, available at: {https://www.riksdagen.se/sv/dokument-lagar/dokument/proposition/tillfalliga-bemyndiganden-i-smittskyddslagen-med_H703155/html#totop}.

90 Ibid.

91 See: {https://www.regeringen.se/48d0af/contentassets/be61e037907843518f7c41d490dd2e78/prop.-2020_21_79.pdf}.

92 See: {https://www.folkhalsomyndigheten.se/the-public-health-agency-of-sweden/communicable-disease-control/covid-19/}.

93 See: {https://svenskforfattningssamling.se/sites/default/files/sfs/2020-07/SFS2020-732.pdf}.

94 Public Order Act, ch. 2, pp. 1–3.

95 See: {https://www.folkhalsomyndigheten.se/the-public-health-agency-of-sweden/communicable-disease-control/covid-19/?exp=69201#_69201}.

96 See: {https://www.regeringen.se/artiklar/2020/03/andring-i-forordning-om-forbud-mot-att-halla-allmanna-sammankomster-och-offentliga-tillstallningar/}.

97 SFS (Stockholm: Svensk Författningssamling, Government Offices of Sweden, 2020), p. 163.

98 See: {https://www.riksdagen.se/sv/dokument-lagar/dokument/svensk-forfattningssamling/forordning-2020163-om-tillfalligt-forbud-mot_sfs-2020-163}.

99 See: {https://www.riksdagen.se/sv/dokument-lagar/dokument/svensk-forfattningssamling/socialtjanstlag-2001453_sfs-2001-453}.

100 SFS (2004), p. 168, available at: {https://www.riksdagen.se/sv/dokument-lagar/dokument/proposition/tillfalliga-bemyndiganden-i-smittskyddslagen-med_H703155/html#totop}.

101 Ibid.

102 See: {https://www.folkhalsomyndigheten.se/the-public-health-agency-of-sweden/communicable-disease-control/covid-19/?exp=69201#_69201}.

103 Summary of the Swedish Covid-19 strategy in English; see: {https://www.youtube.com/watch?v=svlHD2mpk9k&t=351s}.

104 See: {https://www.regeringen.se/regeringens-politik/regeringens-arbete-med-anledning-av-nya-coronaviruset/strategi-med-anledning-av-det-nya-coronaviruset/}.

105 Examples of posters in English; see: {https://www.msb.se/contentassets/48784e70f62a4551925a2924aafd0698/msb0053_verksamheter_affisher_a4_eng_utskrift.pdf}.

106 See: {https://www.youtube.com/watch?time_continue=2&v=roU7RMsrfoI&feature=emb_logo}.

107 See: {https://www.folkhalsomyndigheten.se/smittskydd-beredskap/utbrott/aktuella-utbrott/covid-19/skydda-dig-och-andra/}.

108 See: {https://www.youtube.com/watch?v=svlHD2mpk9k&t=351s}.

109 See: {https://www.youtube.com/watch?v=XltsY72AxW8}.

110 See: {https://www.dn.se/debatt/folkhalsomyndigheten-har-misslyckats-nu-maste-politikerna-gripa-in/}.

111 See: {https://www.youtube.com/watch?v=ZjO9QyNObkE}.

112 See: {https://www.folkhalsomyndigheten.se/nyheter-och-press/nyhetsarkiv/2020/november/start-for-vaccinationer-mot-covid-19-tidigast-i-januari-2021/}.

113 See: {https://www.regeringen.se/regeringens-politik/regeringens-arbete-med-coronapandemin/regeringens-plan-for-avveckling-av-restriktioner/#steg4}.

114 Carl Rådestad and Oscar Larsson, ‘Responsibilization in contemporary Swedish crisis management: Expanding “bare life” biopolitics through exceptionalism and neoliberal governmentality, *Critical Policy Studies* (2018), available at: {https://doi:10.1080/19460171.2018.1530604}; Larsson, ‘The connections between crisis and war preparedness in Sweden'; Veronica Strandh, ‘Crisis volunteerism is the new black?: Exploring the diversity of voluntary engagement in crisis management’, *Risk, Hazards and Crisis in Public Policy*, 10:3 (2019), pp. 311–31, available at: {https://doi.org/10.1002/rhc3.12164}; Sebastian Larsson, ‘In the Name of Society: The Branding of Swedish Civil Security Technologies and Their Exclusionary Effects’ (PhD dissertation, King's College London, 2019), p. 86.

115 Neal, *Security As Politics*.
